# Potential Contributions of miR-200a/-200b and Their Target Gene–Leptin to the Sexual Size Dimorphism in Yellow Catfish

**DOI:** 10.3389/fphys.2017.00970

**Published:** 2017-11-30

**Authors:** Jin Zhang, Wenge Ma, Yan He, Farman U. Dawar, Shuting Xiong, Jie Mei

**Affiliations:** ^1^Key Laboratory of Freshwater Animal Breeding, College of Fisheries, Ministry of Agriculture, Huazhong Agricultural University, Wuhan, China; ^2^Department of Zoology, Kohat University of Science and Technology, Kohat, Pakistan

**Keywords:** miR-200a/b, *leptin*, sexual dimorphism, fasting, sex hormone

## Abstract

Sexual size dimorphism is the consequence of differential expression of sex-biased genes related to feeding and growth. Leptin is known to regulate energy balance by regulating food intake. In order to investigate the molecular mechanism of sexual size dimorphism in yellow catfish (*Pelteobagrus fulvidraco*), the expression of *leptin* (*lep*) and its functional receptor (*lepr*) were detected during larval development. Both *lep* and *lepr* have lower expression in males than in females during 1–4 weeks post hatching. 17a-Methyltestosterone (MT) treatment resulted in decreased expression of *lep* and *lepr* in both male and female larval fish. Interestingly, the mRNA levels of *lep* and *lepr* in juvenile male were significantly decreased compared with juvenile female during short-term fasting periods. *Lep* was predicted to be a potential target of miR-200a and miR-200b that had an opposite expression pattern to *lep* in male and female larvas. The results of luciferase reporter assay suggested that *lep* is a target of miR-200a/-200b. Subsequently, male hormone and fasting treatment have opposite effects on the expression of miR-200a/-200b and *lep* between males and females. In summary, our results suggest that sexual size dimorphism in fish species is probably caused by the sexually dimorphic expression of *leptin*, which could be negatively regulated by miR-200a/-200b.

## Introduction

Sexual size dimorphism is a systematic difference in size, shape, color, physiology and behavior between male and female individuals of the same species in vertebrates. The sexual size dimorphism that is due to different growth rate between male and female individuals has been found in many cultivated fish species, such as tilapia (*Oreochromis niloticus*) (Beardmore et al., 2001), rainbow trout (*Oncorhynchus mykiss*) (Bye and Lincoln, 1986) and yellow catfish (*Pelteobagrus fulvidraco*) (Gui and Zhu, 2012; Mei and Gui, 2015). Although, studies have been conducted to elucidate the sexual size dimorphism in fish, the exact molecular mechanism underlying is still unclear (Mei and Gui, 2015).

Growth hormone (GH)/insulin-like growth factor (IGF) is crucial in regulating somatic growth in vertebrates (Duan, 1998; Perez-Sanchez and LeBail, 1999; Reinecke, 2006). Ghrelin is a potent stimulator of growth hormone (GH) from pituitary and has been recognized to promote food intake and body weight gain (Tschöp et al., 2000; Nakazato et al., 2001). As an anorexigenic peptide hormone, Leptin circulates to maintain energy homeostasis by balancing feeding and energy expenditure in the body of vertebrates (Ahima and Flier, 2000; Arora, 2008). Moreover, Leptin could regulate GHR and IGFs expression to coordinate somatic growth with nutritional state (Won et al., 2016).

The miRNAs are small non-coding RNA molecules that play predominantly inhibitory regulatory roles to gene expression by binding to the 3′untranslated region (3′UTR) of target mRNAs (Bartel, 2004; Derghal et al., 2017). MiR-200 family is highly conserved in vertebrates, including miR-200a/200b/200c/429a/429b/141, which are widely expressed in many tissues, such as pituitary gland, testes, prostate gland, ovary, breast, and liver (Landgraf et al., 2007). It is widely known as an inhibitor of the epithelial-to-mesenchymal transition and tumor suppressor (Griffiths-Jones et al., 2006; Becker et al., 2015; Trümbach and Prakash, 2015). In zebrafish, miR-200 family members have been shown to control body size by reducing the expression of *GH, GHRa, GHRb, IGF1*, and *IGF2a* during embryo development (Jing et al., 2015). Research in mice suggested that miR-200a was involved in the regulation of *leptin* and insulin expression in the hypothalamus (Crépin et al., 2014; Derghal et al., 2017).

Yellow catfish is a distinctive species for sexual size dimorphism, in which males grow much faster than females. Some reports find that sexually dimorphic expression genes were identificated in yellow catfish transcriptome (Lu et al., 2014, 2015; Wu et al., 2015). Our previous study demonstrated that sex differences in the expression of GH/IGF signaling genes and *ghrelin*/*GHSR* might be involved in the sexual size dimorphism in yellow catfish. The sex hormones, 17-Alpha-methyltestosterone (MT) and 17-alpha-ethinyl estradiol (EE2) could alter expression of these genes related to body growth (Ma et al., 2016; Zhang et al., 2016). In this study, we investigated expression changes of miR-200a/-200b and their target gene *leptin* during larval development, under the effects of fasting and sex hormone treatment. Our results demonstrate potential contributions of miR-200a/-200b and their target gene *leptin* to the sexual size dimorphism in yellow catfish.

## Materials and methods

### Animals and samples

Ten male and ten female two-year-old sexually mature yellow catfish were obtained for artificial fertilization from our breeding center at Huazhong Agricultural University, China. Different stages of larval and juvenile fish were treated and sampled in accordance with the guiding principles approved by the institution animal care and use committee of Huazhong Agricultural University (Ethical Approval No. HBAC20091138; Date: 15 November 2009).

To investigate the effects of fasting on gene expression, short-term fasting experiments were designed. As mentioned in our previous reports (Zhang et al., 2016), yellow catfish were cultured in six glass tanks and in 25–26°C aerated water with a natural photoperiod in standard 24 hr time clock. In brief, all fish were fed at 8:00 and 16:00 using commercial blood worm for 2 weeks. A group of juvenile fish was fasted, while others were fed as above. Fifteen fish (five from each group) were randomly collected on specific time period (8:00, 8:30, 9:30, 11:00, 14:00). The fish was sampled at 8:00 a.m. to detect gene expression prior to normal feeding time.

To evaluate the effects of sex hormone on gene expression, 4-day-old larvae (size: 4 ± 0.3 mm) were randomly selected after artificial fertilization. The fish were separately cultured in two groups and maintained at 25–26°C. One group was fed thrice a day with filtered artemia that was firstly soaked in 100 μg/L 17a-methyltestosterone (MT) for 1 h. The control group was fed with filtered artemia without MT treatment. Samples were collected weekly for 4 weeks. The whole fish (larvae and juveniles) were sampled in all experiments because the fish were too small to collect the specific tissues. The tail of each fish was cut down for sex identification using sex-linked markers as previously described (Dan et al., 2013), and the rest of the fish (head and trunk) was frozen in liquid nitrogen and stored at −80°C until RNA extraction. The experimental condition and method were the same as two previous studies (Ma et al., 2016; Zhang et al., 2016).

### Total RNA extraction and cDNA synthesis

The extraction of total RNAs from yellow catfish samples was conducted by TRIzol (Invitrogen, USA) according to the manufacturer's instruction. Each sample was collected from 5 individuals. RNA quality and quantity were determined by A260 measurement (NANODROP2000, Thermo). RNA integrity was assessed by electrophoresis. RNA was diluted to 200 ng/μL with RNase-free deionized water. Subsequently, the total RNA was reverse transcribed into cDNA using PrimeScript™ RT reagent Kit with gDNA Eraser (Stratagene, Takara).

### Quantitative analysis of gene expression by qRT-PCR

The expression levels of genes were examined using quantitative fluorescent RT-PCR (Bio-rad, USA). The qRT-PCR was accomplished using β-actin as the internal control. Primers were designed by Primer Premier 5.0 software and listed in Table 1. The protocol of qRT-PCR and analysis method were used as described, and the relative expression of each gene was analyzed using 2^−ΔΔCt^ method (Zhang et al., 2016). The 20 μL reaction cocktail included 10 μL 2 × SYBR green master mix (Bio-rad, USA), 0.5 μL (10 μM) of each primers (forward and reverse), 2 μL cDNA template and 7 μL double distilled water. The reaction conditions were as follows: 95°C for 30 s followed by 40 cycles at 95°C for 10 s, 58°C for 10 s and 72°C for 15 s and concluding with a single elongation step at 72°C for 5 min (Bio-rad, USA). Melt curve analysis was performed to verify single product generation at the end of the assay. The amplification efficiencies were 1.09, 0.82, 0.98, 1.01, 1.02, 1.03 for *lep, lepr*, β*-actin, miR-200a, miR-200b*, and *5.8s* that were calculated according to a previous description (Sinha et al., 2015). Amplicons were verified by sequencing (TSINGKE, Beijing). All experiments and measurements were performed in triplicate.

**Table 1:** The primers for qRT-PCR.

**Primers**	**Sequences(5′-3′)**	**Applications**	**Size of the products (bp)**	**Genbank Accession**
Leptin-F	ACTTCCAGCGAGTCCTTC	qPCR	209	JQ288727
Leptin-R	CAGTCTGTCCAGAGCCAC	qPCR	209	
Leptin receptor-F	AGCCAATCAGAGCCTTAG	qPCR	160	JX118825
Leptin receptor-R	TACATTCGCTTGTTCGTC	qPCR	160	
miR-200a-F	GCGCTAACACTGTCTGGTAA	qPCR	71	MG383687
miR-200a-R	GTGCAGGGTCCGAGGT	qPCR	71	
miR-200b-F	GCGCTAATACTGCCTGGTAA	qPCR	73	MG383688
miR-200b-R	GTGCAGGGTCCGAGGT	qPCR	73	
β-actin-F	TCCCTGTATGCCTCTGGTCGT	qPCR	179	EU161065
β-actin-R	AAGCTGTAGCCTCTCTCGGTC	qPCR	179	
5.8s-F	TCTTAGCGGTGGATCACTCG	qPCR	51	GQ376028
5.8s-R	AGCTGGCTGCGTTCTTCAT	qPCR	51	

### Cell transfection and luciferase reporter assay

3′UTR fragments of *leptin* gene, which contains one putative miR-200a/-200b binding site was inserted into the pmir-GLO plasmid (Promega). Then, the binding site of miR-200a (AGTGTT) in the constructed wild-type plasmid was replaced with GACACG by site-directed mutagenesis (Mei et al., 2011). MUT represented the mutation of predicted binding site of miR-200a/-200b in *leptin* gene. Hela cells were transiently transfected with plasmid of wild-type (25 ng) or mutant (25 ng) and miRNA mimics (50 nM) or negative control (50 nM) per 24-well using DharmaFECT transfection reagent (Dharmacon). Twenty-four hours post transfection, luciferase activity was measured using Dual Luciferase reporter assay system (Promega). Relative reporter activities were determined by normalizing Firefly activity to Renilla activity.

### Statistical analysis

Each result and figure was from three independent experiments as biological replicates. Data are represented as the mean ± SEM from three independent experiments. Differences in dependent variables were analyzed in a two-way ANOVA according to general linear modeling using treatment and time as fixed factors. Data was tested for homoscedasticity and normality. A probability (*P* < 0.05) was considered statistically significant. ^*^P < 0.05, ^**^P < 0.01, and ^***^P < 0.001.

## Results

### Expression of *leptin, leptin* receptor and miR-200a/-200b during larval development

**Figure 1:**
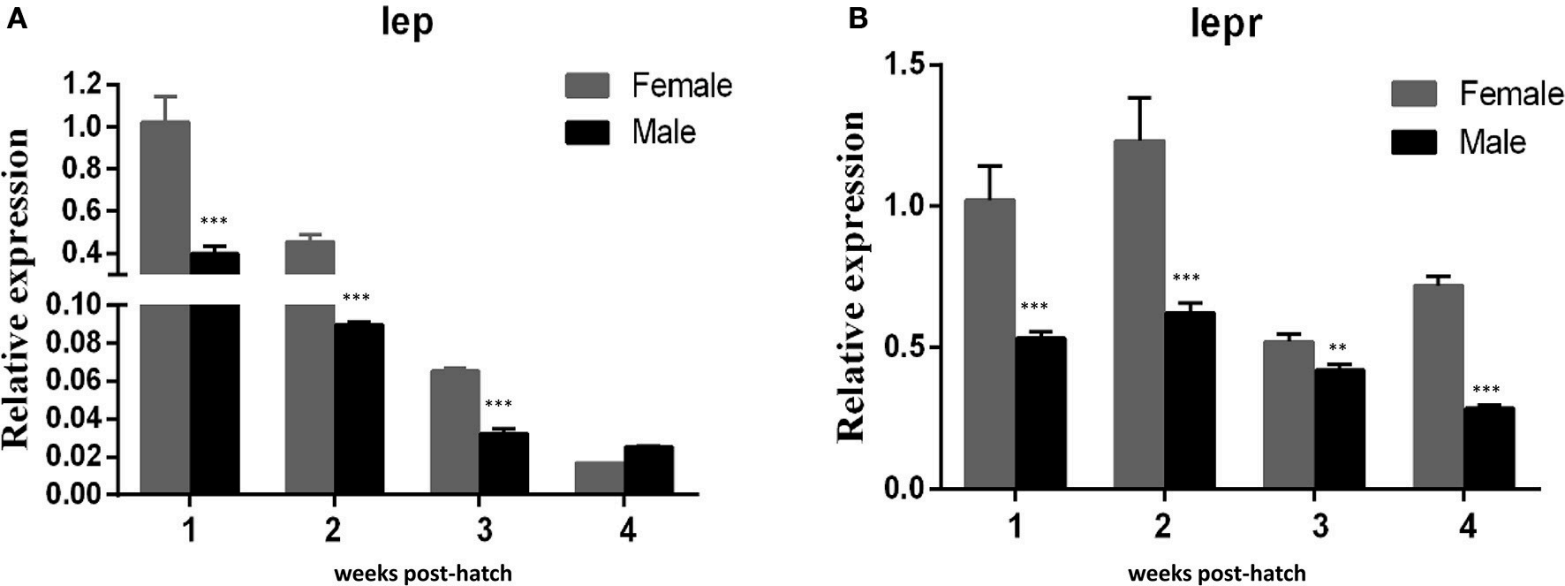
Expression of *lep* and *lepr* in juvenile fish among 1–4 weeks post hatching. **(A,B)** Indicated the relative expression levels of *lep* and *lepr* gene during juvenile fish, respectively (^**^P < 0.01 and ^***^P < 0.001).

**Figure 2:**
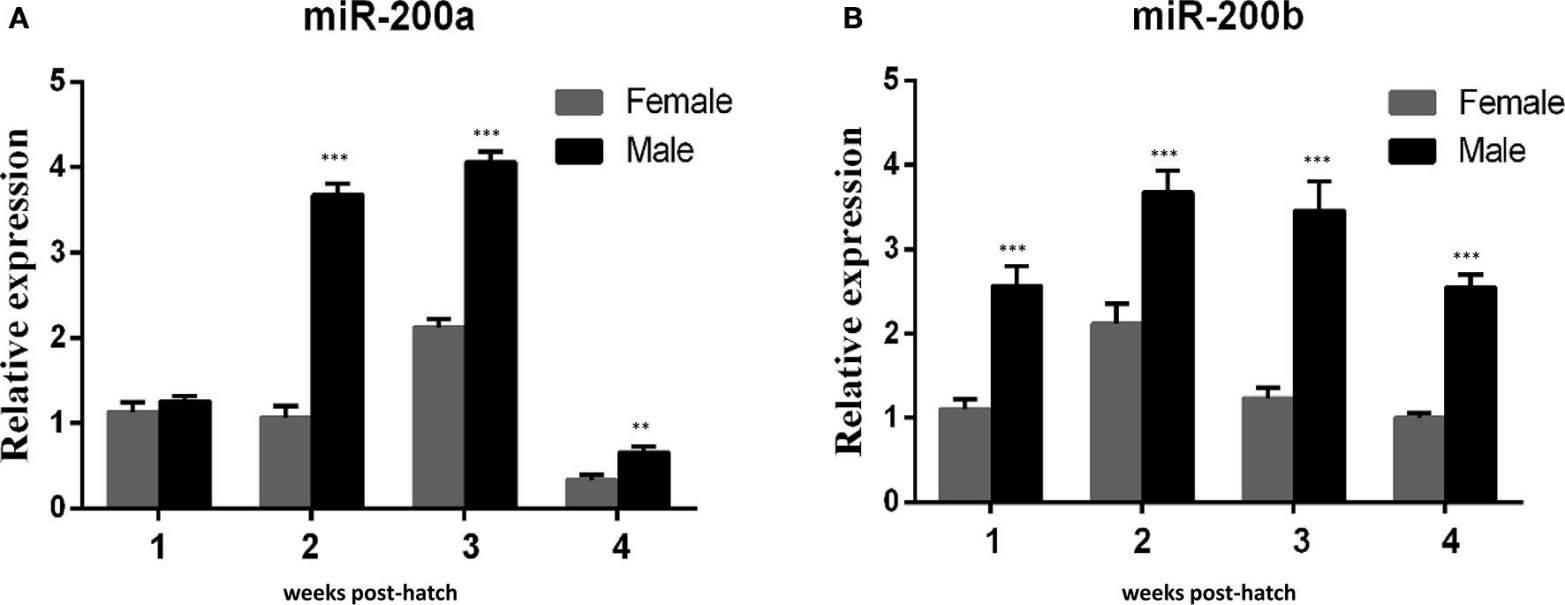
Expression of miR-200a and miR-200b in juvenile fish among 1–4 weeks post hatching. **(A,B)** indicated the relative expression levels of miR-200a and miR-200b during juvenile fish, respectively (^**^P < 0.01 and ^***^P < 0.001).

QRT-PCR was performed to detect the expresson of *lep* (JQ288727), *lepr* (JX118825) and miR-200a/-200b during larval development in yellow catfish. *Lep* was expressed significantly lower in male larvae than in female during the stage of 1–3 weeks post hatching (wph), whereas there was no significant expression difference at 4 wph (Figure 1A). In addition, *lepr* was expressed significantly lower in male larvae than female during 1–4 wpf (Figure 1B). In contrast, the expression of miR-200a was significantly higher in male larvae than in female during the stage of 2–4 wph, whereas there was no significant expression difference at 1 wph (Figure 2A). The expression of miR-200b was significantly higher in male larvae than in female during 1–4 wph in yellow catfish (Figure 2B).

### *Lep* is a potential target of miR-200a/-200b

**Figure 3:**
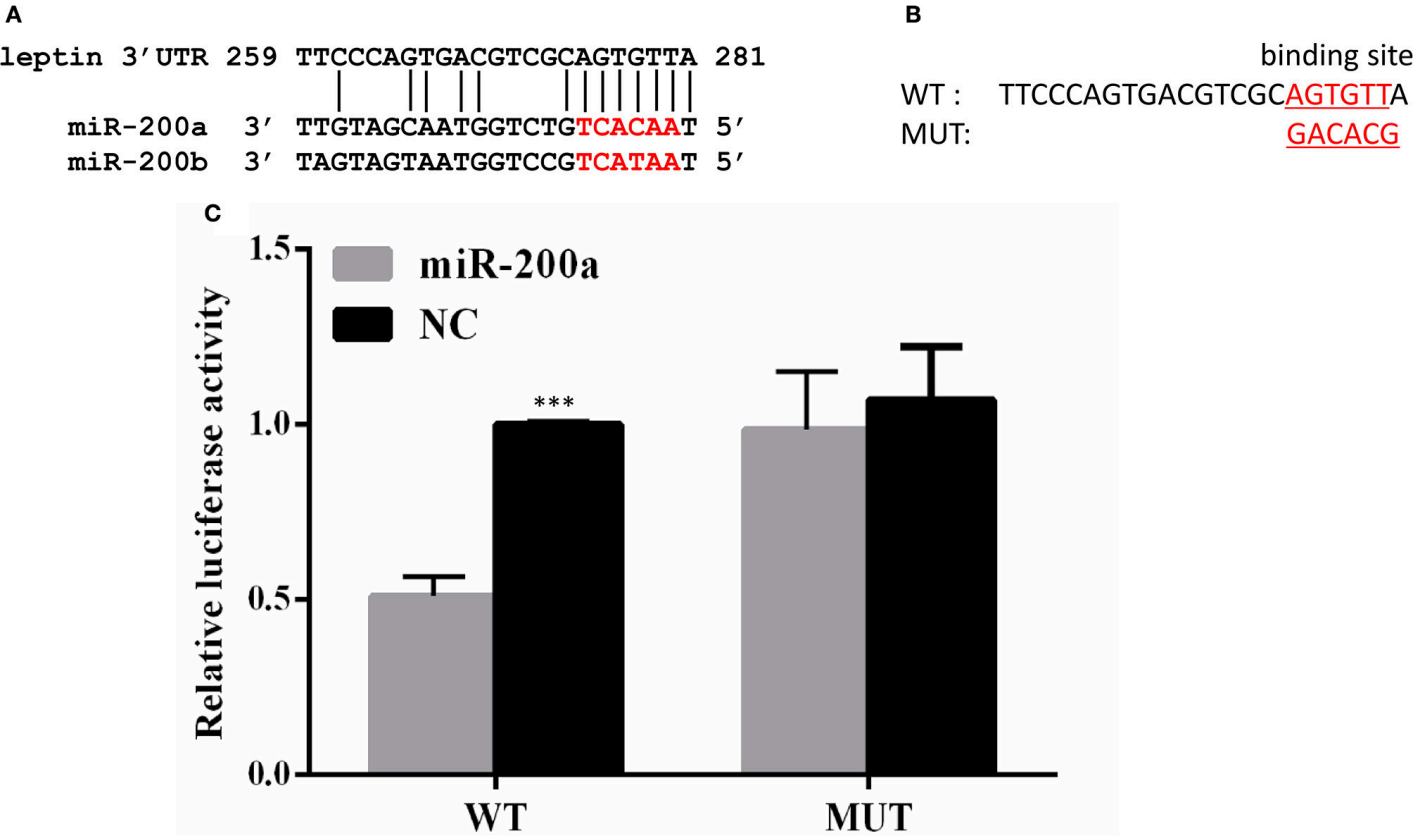
*Lep* is a target of miR-200a. **(A)** Conserved miR-200a seeding sequence and its binding site in the leptin 3′UTR are marked in red. **(B)** The sequence information of the putative *lep* 3′UTR binding site in wild type (WT) and mutant (MUT) were showed. **(C)** miR-200a suppressed the activity of pmirGLO-*lep* 3′UTR plasmid, but not mutant vector. The Firefly activity was normalized to Renilla expression, which was used as a control for transfection efficiency. Luciferase assays were performed in triplicate and were representative of 3 independent experiments. This experiment was carried on in Hela cell line. NC, negative control. The asterisk indicates the significant differences of gene expression between different groups (^***^P < 0.001).

A binding site for miR-200a and miR-200b was detected in 3′UTR of *lep*, and the seeding sequences of miR-200a and miR-200b are the same (Figure 3A). To determine whether *lep* is a direct target gene of zebrafish miR-200a/-200b, we performed a luciferase reporter assay by linking the 3′UTR of *lep* to the C-terminus of Firefly luciferase present in pmirGLO vector. PmirGLO/3′-UTR (WT) and its mutant vector (MUT) (Figure 3B) were co-transfected with miR-200a or control microRNA mimic into Hek 293T cells. Luciferase reporter assay showed that miR-200a significantly repressed the luciferase activity of leptin 3′UTR-pmirGLO, whereas mutation in the binding site abrogated this repression (Figure 3C).

### Sexually dimorphic expression of *Lepr* and miR-200a/-200b during fasting

**Figure 4:**
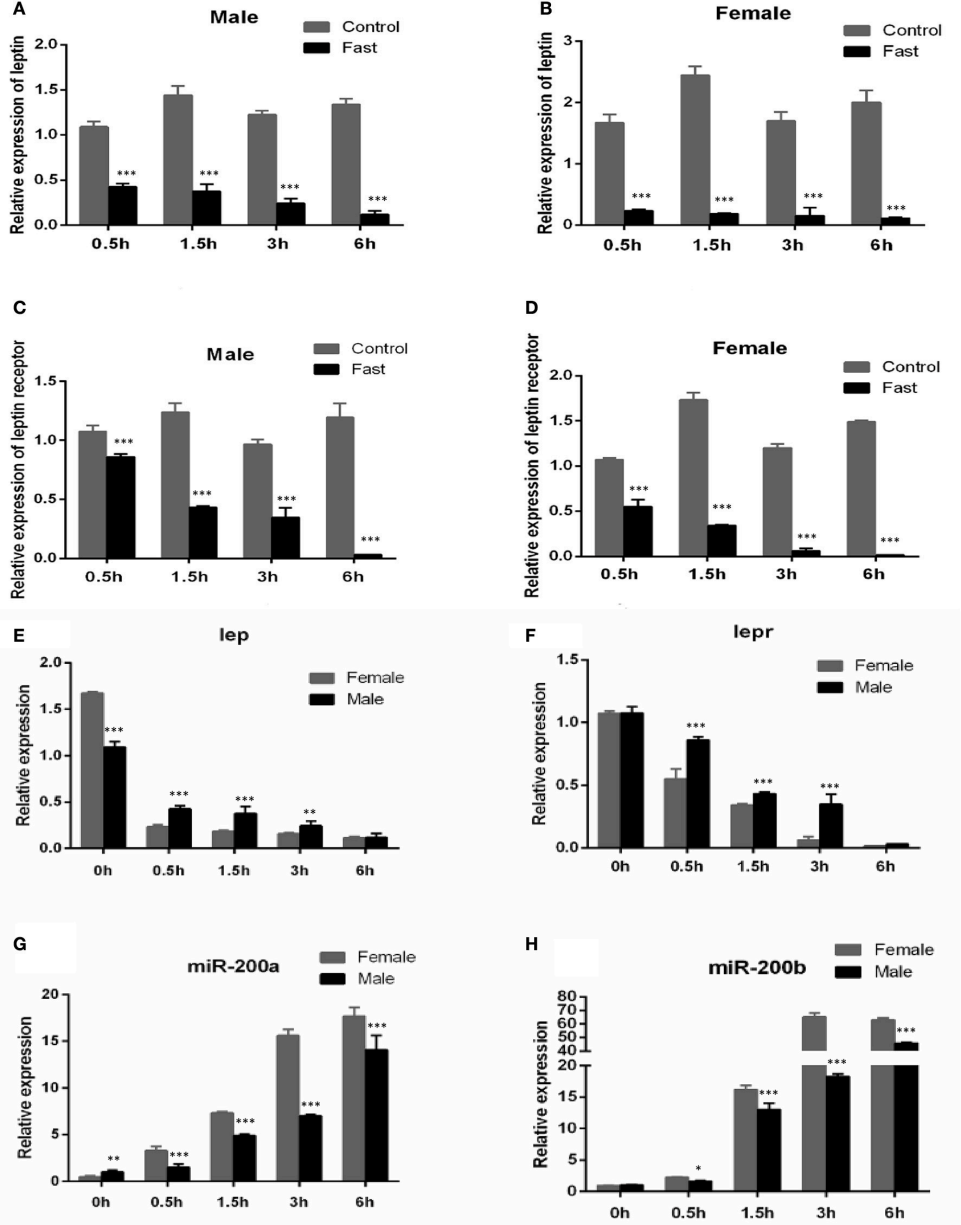
Effects of fasting on the expression of *lep, lepr* mRNA and miR-200a/b during juvenile growth in yellow catfish. **(A–D)** showed the effects of short-term fasting on the expression of *lep* **(A,B)** and *lepr* **(C,D)** mRNA during growth in female and male juvenile yellow catfish respectively. Fast, fasting group; Control, control group. **(E–H)** showed the effects of short-term fasting on the expression of *lep* **(E)**, *lepr* **(F)**, and miR-200a **(G)** and miR-200b **(H)** mRNA in male and female juvenile yellow catfish, respectively. The asterisk indicates the significant differences of gene expression between different groups (^*^P < 0.05, ^**^P < 0.01, and ^***^P < 0.001).

As shown in Figure 4, the expression of *lep* (Figures 4A,B) and *lepr* (Figures 4C,D) were significantly decreased in short-term fasting and had very low expression levels at 6 h post fasting, both in male and female yellow catfish. Compared with the expression in fasted females, the mRNA levels of *lep* (Figure 4E) and *lepr* (Figure 4F) were significantly higher in fasted male yellow catfish during 0.5–3 h fasting. Moreover, the expressions of miR-200a and miR-200b in fasted males were significantly lower than the expression in fasted females during 0.5–6 h fasting (Figures 4G,H).

### Sexually dimorphic expression of leptin and miR-200 after hormone treatment

**Figure 5:**
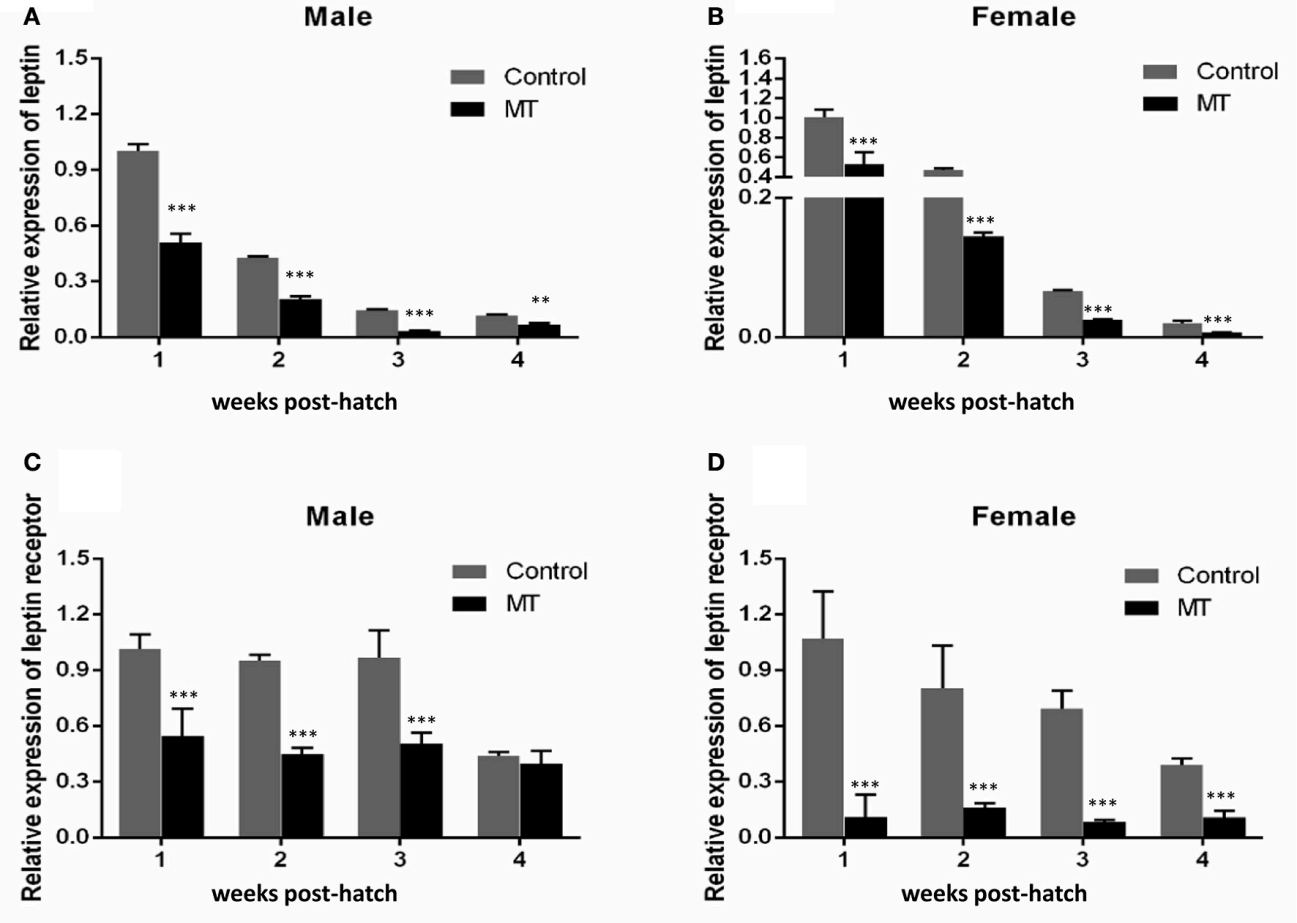
Effects of MT on the expression of *lep* and *lepr* mRNA during juvenile growth in male and female yellow catfish. **(A–D)** showed the expression of *lep* and *lepr* mRNA respectively. MT, MT treatment group; Control, control group. The asterisk indicates the significant differences of gene expression between the females and males (^**^P < 0.01 and ^***^P < 0.001).

**Figure 6:**
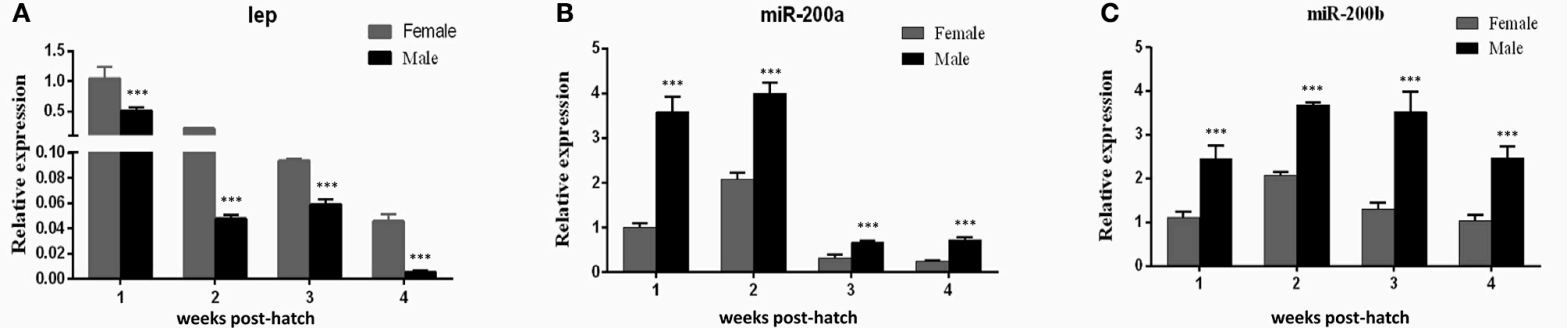
Different effects of MT on the expression of *lep* and miR200a/b mRNA during juvenile growth between male and female yellow catfish. **(A–C)** showed the effects of MT on the expression of *lep* **(A)** and miR-200a **(B)** and miR-200b **(C)** mRNA in male and female juvenile yellow catfish, respectively. The asterisk indicates the significant differences of gene expression between the females and males (^***^P < 0.001).

Our previous study indicated that sex differences in the expression of *ghrelin* induced by MT might be involved in the sexual size dimorphism in yellow catfish (Zhang et al., 2016). Ghrelin and leptin play important roles in the regulation of food intake and body weight (Klok et al., 2007). Therefore, the mRNA levels of *lep* (Figures 5A,B) and *lepr* (Figures 5C,D) were analyzed and suggested that MT treatment was able to significantly reduce expressions of *lep* and *lepr*, both in male and female yellow catfish. Compared with each other, MT treatment resulted in a greater decrease of *lep* expression in males than in female yellow catfish (Figure 6A). In contrast, we detected a greater increase of miR-200a and miR-200b expression in males than in female yellow catfish after MT treatment (Figures 6B,C).

## Discussion

**Figure 7:**
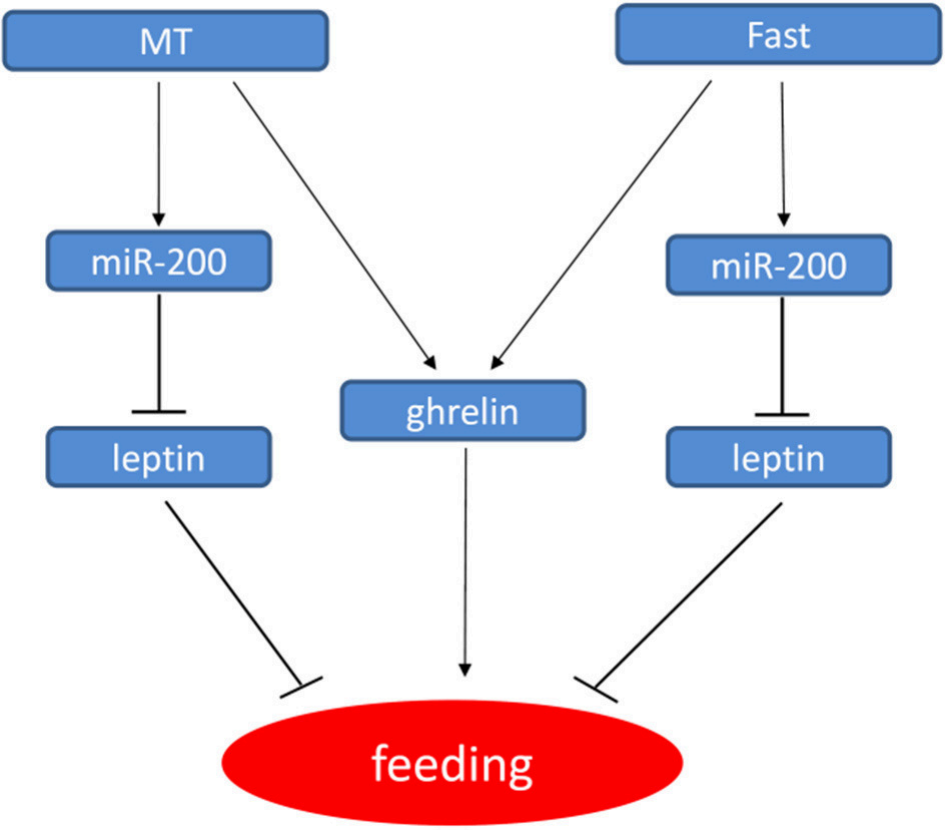
Model for the regulatory networks of feeding in yellow catfish involved miR-200/leptin. In yellow catfish juvenile, miR-200 regulate feeding by directly repressing leptin gene. Further, miR-200 and ghrelin genes are up-regulated by MT and fast treatment, thereby resulting in the down-regulation of leptin and leptin receptor, thus forming a regulatory network.

Sexual difference in growth performance has been observed in many cultivated fish species including yellow catfish, but the molecular mechanism of sexual size dimorphism is still unclear. Several studies have indicated that sexual dimorphism in vertebrates, including fish species are the consequence of sex-biased gene expression and are controlled by multiple critical genes during growth and development (Williams and Carroll, 2009). Our previous studies (Ma et al., 2016; Zhang et al., 2016) have showed that sex difference in the expression of *ghrelin* and *GHSR* may be involved in sexual size dimorphism by regulating feeding and GH/IGF signaling in yellow catfish, and both male hormone and fasting could increase the expression of ghrelin (Figure 7). Leptin and Ghrelin are two hormones that have been recognized to play a major role in energy balance. Leptin generally suppresses food intake and thereby inducing weight loss. Ghrelin and GHSR are fast-acting hormones regulating meal initiation. Leptin and Ghrelin stimulate and suppress hypothalamic neurons, resulting in anorexic or orexic effects on energy balance, respectively (Klok et al., 2007). As an anti-appetite peptide hormone, Leptin has been proved to regulate growth axis component transcripts and correlated with somatic growth with nutritional state (Won et al., 2012). However, there is no report about the correlation of Leptin with the sexual size dimorphism.

To prevent excessive fat accumulation, Leptin can suppress appetite (Ahima and Flier, 2000). In the present study, *leptin* expressed much lower in male larvae than in female larvae during 1–3 weeks post hatching (Figure 1), which may explain why male yellow catfish grow much faster than females. In addition, male hormone (MT) could reduce the expression of *lep* and *lepr* in both male and female larvae (Figure 5). Shen et al. reported that oral administration of MT at different doses did not significantly change the ratio of males compared to the control group, but produced intersex fish. Their results demonstrated that MT may affect gonadal development and subsequently promot body growth (Shen et al., 2015). Probably, there is a potential interaction between sex and food intake. Leptin can regulate gonadotropic axis, combined with other promoting factors in rainbow trout (Weil et al., 2003). Yellow catfish *leptin* is expressed in many tissues, such as liver, ovary, mesenteric fat, and spleen (Gong et al., 2013).

The nutritional status is positively correlated with growth performance. In mammals, leptin increases during feeding and promotes satiety and induces lipolysis (Ahima and Flier, 2000). In contrast, leptin decreases during fasting because of the depletion of adipose stores (Harris et al., 1996; Sandoval and Davis, 2003). *Leptin* has been cloned in many fish species, such as rainbow trout and salmonid, and it plays important roles in fish somatic growth (Kling et al., 2012; Trombley et al., 2012). In hybrid striped bass, hepatic *leptin* mRNA expression is sensitive to the nutritional states, declining during fasting catabolic states and increasing under anabolic conditions (Won et al., 2012). Similar to the present study, a recent study indicated that expression of a leptin-like peptide decreases with fasting in green sunfish (Johnson et al., 2000). Administration of exogenous Leptin effectively suppressed the appetite and feeding in goldfish (Volkoff et al., 2003; de Pedro et al., 2006), striped bass (Won et al., 2012), rainbow trout (Murashita et al., 2008), and African clawed frog (Crespi and Denver, 2006). However, different from in mammals, there was no unified correlation between Lep levels and fasting condition in fish species. For example, *leptin* had an increase trend during fasting in multiple teleosts (Copeland et al., 2011; Gorissen and Flik, 2014), such as in fine flounder, anadromous Arctic charr and rainbow trout (Kling et al., 2009; Fuentes et al., 2012; Jørgensen et al., 2013). In some other studies, fasting was shown to either stimulate leptin or had little effect on leptin expression in goldfish (Volkoff et al., 2003; Tinoco et al., 2012) and striped bass (Huising et al., 2006; Won and Borski, 2013), which might be due to different experimental situations. In our present study, *leptin* gene expression significantly decreased after fasting both in male and female yellow catfish (Figure 4), suggesting that the adipostatic model for Leptin in mammals may also be applied in yellow catfish.

Mouse miR-200a has been shown to regulate expression of leptin and insulin in the hypothalamus (Crépin et al., 2014; Derghal et al., 2017). In fish species, zebrafish miR-200 family regulate body size by reducing the expression of *GH, GHRa, GHRb, IGF1*, and *IGF2a* during embryo development (Jing et al., 2015), while rainbow trout miR-200 family members abundantly expressed in the somatic tissues including stomach. And some target genes of miR-141/-200a/-429 were also predicted, which provide a clue to study functions of miR-200 family (Salem et al., 2010). Here, we reported that miR-200a/-200b might play some roles in sexual size dimorphism by targeting *lep* gene in yellow catfish. Subsequent male hormone treatment and fasting experiments showed that miR-200a/-200b negatively regulate *lep* gene, and the regulation is different between male and female yellow catfish. We described the model how male hormone and fasting stress regulate the expression of *ghrelin*, miR-200a/b and *leptin*, and further food intake (Figure 7). The expression of miR200a/-200b was induced after MT treatment to inhibit *leptin* mRNA level. The decreased leptin level subsequently up-regulates feeding signal. In summary, our results suggest that sexual size dimorphism in yellow catfish is partially caused by the sex difference in the expression of *lep* that could be negatively regulated by miR-200a/-200b.

## Author contributions

JZ, YH, and JM conceived and coordinated the study and wrote the paper. JZ and WM designed, performed and analyzed the experiments shown in Figures. SX and FD provided technical assistance and contributed to the preparation of the figures. All authors reviewed the results and approved the final version of the manuscript.

### Conflict of interest statement

The authors declare that the research was conducted in the absence of any commercial or financial relationships that could be construed as a potential conflict of interest.
